# Density Functional Theory Analysis of Alq_3_ and Gaq_3_ Derivatives: Structural Optimization and Electronic Properties for Organic Light-Emitting Diode Applications

**DOI:** 10.1021/acsomega.5c03335

**Published:** 2025-08-23

**Authors:** Huai-Wen Tsai, Chung-Chieh Tai, Wen-Ken Li

**Affiliations:** † Department of Electrical Engineering, Chien Hsin University of Science and Technology, Taoyuan 320678, Taiwan; ‡ Department of Semiconductor and Electro-Optical Technology, 63364 Minghsin University of Science and Technology, Hsinchu 30401, Taiwan; § Department of Mechanical Engineering, 34900 Chung Yuan Christian University, Taoyuan 32023, Taiwan

## Abstract

This study employs computational quantum mechanics to investigate the impact of molecular and electronic structures on the optical properties of organic light-emitting diodes (OLEDs). First-principles calculations based on density functional theory (DFT) and time-dependent density functional theory (TD-DFT) were used to analyze Mq3 and Mq2p (M = Al or Ga) and their derivatives, where one quinoline ligand was replaced with picolinate and CH/N substitutions were introduced in the qa and qc ligands. The molecular structures were optimized using time-independent DFT, while electronic excitation energies were determined using time-dependent DFT. Based on the optimized ground-state structures, key molecular properties, including bond length, bond angle, dipole moment, band gap, electron cloud energies, ionization energy, electron affinity, and reorganization energy, were systematically computed. Additionally, absorption and emission spectra were examined, revealing tunable Stokes shifts. The results indicate that Gaq_3_ derivatives exhibit superior structural stability and improved hole-blocking and electron injection capabilities compared to Alq_3_. These findings offer valuable guidance for designing superior OLED materials, potentially enhancing light emission and electronic transport.

## Graphical abstract

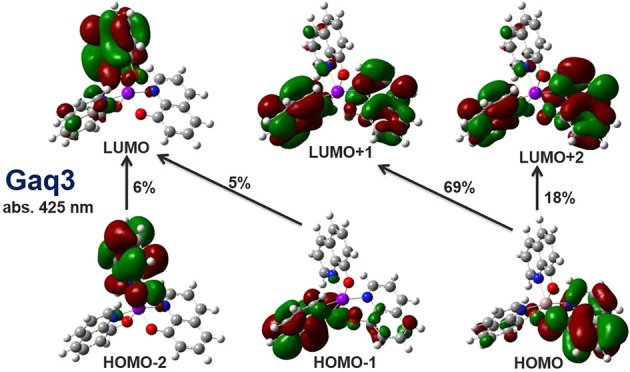

## 1 Introduction

The emissive electroluminescent layer of an organic light-emitting diode (OLED) is a film made of organic compounds that emit light in response to an electric current.^1,2^ OLEDs are the future of flat panel display technology because they are as thin as their liquid-crystal diode (LCD) counterparts but offer more vibrant colors.^3^ In addition, OLEDs can be fabricated on a flexible substrate for use in flexible electronics. OLEDs are made of luminous tris (8-hydroxyquinolinato) aluminum (Alq3), which has good stability and longevity. Relative to LCDs, OLEDs have the following advantages.^4,5^ (1) They are self-luminous and thus require no external backlight (in addition to being lighter and thinner). (2) They have wider viewing angles that can reach 170°. (3) They are more power efficient because of their simple panel structure and correspondingly high light utilization. (4) They have a high resolution; a single pixel can be smaller than 0.3 mm^2^. (5) They exhibit a shorter response time of approximately 0.01 ms (approximately 1000 times faster than that of LCDs); this also eliminates the LCD disadvantage of motion blur. (6) They offer greater temperature and shock resistance. (7) Their design is flexible; that is, small molecules can be deposited on a flexible plastic substrate and used to make a flexible panel.

In 1987, Tang and VanSlyke fabricated an OLED by applying the vacuum deposition method to plated Alq3.^6,7^ Tris­(8-hydroxyquinoline) metals (Mq3, M = Al, Ga, or In) have two geometric isomers, namely, the meridional (mer) and facial (fac) isomers. Their crystals undergo several polymorphous modifications, where the mer isomers are in the α, β, and ε phases and fac isomers are in the γ and δ phases.^8,9^ An α-phase crystal (Alq3 or Gaq3) can be converted to the δ phase through thermal treatment,^10,11^ and mer-Alq3 is more structurally stable than fac-Alq3. Therefore, most applications of Alq3 use mer-Alq3.^12,13^ The electron cloud of Alq3 is distributed on the quinoline ligands, where the filled π orbitals (highest occupied electron clouds [HOMOs]) are located on the phenoxide side of the quinolone ligands and the unfilled π* orbitals (lowest unoccupied electron clouds [LUMOs]) are located on the pyridine side.

Gaq3 structures can have greater stability and luminous efficiency than those of Alq3.^14,15^ The analogue and optical properties of the OLED bands of Alq3 were studied using density functional theory (DFT), and the central metal ions of Al^3+^ and Ga^3+^ were replaced with Alq3 and Gaq3, respectively. Subsequently, various derivatives have been constructed and analyzed. Zhang and Frenking^16,17^ investigated the molecular bonding and electron cloud distribution to optimize the structures of Alq3 and Gaq3. Using ab initio analyses and DFT with different base functions, Gahungu and Zhang^18^ investigated the properties of Gaq3, such as its lowest-energy structure and electronic characteristics, by employing DFT and time-dependent DFT (TD-DFT) to simulate Gaq3 and Alq3 and replacing CH bonds with N atoms to obtain six distinct derivatives. They discovered that the absorption and radiation wavelengths differed among the derivatives and that the light emission wavelength could be tuned. Gahungu et al.^19^ expanded on this by examining CH/N substitutions and picolinate (p) ligand modifications in Alq_3_ derivatives, showing that substituent positioning tunes emission wavelengths and affects HOMO–LUMO levels. Liu et al.^20^ demonstrated that modifying the indium tin oxide anode with Ni_2_O_3_ or MoO_3_ significantly improves hole injection efficiency and enhances the current efficiency of blue-emitting OLEDs. Their work emphasized that band alignment at the anode/organic interface is crucial for optimizing carrier injection, leading to the development of OLEDs with improved efficiency and stability. In 2012, using hybrid Heyd–Scuseria–Ernzerhof DFT as their theoretical basis, Bisti et al.^21^ used core level and valence band photoemission spectroscopy to evaluate tris (8-hydroxyquinolinato) erbium­(III) (Erq3) and tris (8-hydroxyquinolinato) aluminum (Alq3). Gorter et al.^22^ used inkjet printing to create multilayered small-molecule OLED devices. Gaq3 had a higher glass transition temperature (*T*_g_ = 182 °C) than did Alq3 (*T*_g_ = 173 °C), suggesting it has a stronger dipolar interaction due to a Ga^3+^ cation effect.^23^ Painuly et al.^24^ investigated the thermal-induced transformation of α-Alq_3_ to ε-Alq_3_ and reported that this phase transition leads to a blue shift (∼18 nm) in emission wavelength, an increase in band gap, and reduced thermal stability. Their study further revealed that ε-Alq_3_ exhibits a larger band gap and lower thermal stability compared to that of α-Alq_3_, which can influence its performance in OLEDs. Vergara et al.^25^ explored the fabrication of hybrid Alq_3_-based films doped with tetracyanoquinodimethane (TCNQ) for use in photoconductive devices. Their study demonstrated that the incorporation of TCNQ into Alq_3_ enhances charge transport and reduces the optical band gap, making it a promising candidate for OLEDs and other optoelectronic applications.

In this study, we construct and analyze molecular models of Alq_3_, Gaq_3_, and their derivatives, where one of the quinoline (q) ligands is replaced with a picolinate (p) ligand. Unlike previous studies that primarily focused on the structural and optical properties of Alq_3_ and Gaq_3_ individually, this work provides a comprehensive investigation into the effects of CH/N substitutions at various positions within the picolinate-modified derivatives. Using DFT and TD-DFT, we systematically evaluate key electronic and optical properties, including total ground-state energy, dipole moment, absorption and emission spectra, HOMO–LUMO energy levels, band gap, ionization energy, and electron affinity. This study not only quantifies the stability and electronic transitions of these derivatives but also elucidates the tunability of their optical properties based on substitutional modifications. By establishing correlations between molecular structure and optoelectronic performance, our findings offer valuable insights for the design of next-generation OLED emitters and electron transport materials.

## 2 Model Construction and Simulation Methods

### 2.1 Theoretical Background

To investigate the electronic properties of a multielectron system, we employed density functional theory (DFT) for first-principles calculations. A key aspect of electron–electron interactions in such calculations is the exchange–correlation term, which is modeled by using various semiempirical approaches. The most commonly used model was proposed by Becke, Lee, Yang, and Parr (the eponymously named BLYP model).^26^ Geometric optimization of the ground state was performed using Becke’s three-parameter hybrid functional combined with the Lee, Yang, and Parr (LYP) correlation functional (denoted as B3LYP^27^) and the 6–31G­(d)^28^ basis set. For comparison, we used the Hartree–Fock theory combined with the 3–21+G­(d,p) basis set, which previously demonstrated sufficient accuracy in the study of Alq3. The first excited-state structure (S_1_) was optimized using ab initio single-excitation configuration interaction (CIS),^29^ and the absorption and emission energies were calculated using TD-DFT B3LYP/3–21+G­(d,p),^30^ which has yielded accurate estimates in studies of Alq3 and its analogues, accounting for electron correlation. All quantum chemical calculations were performed using the Gaussian 03 C2 package,^31^ and GaussView 4.1.2 was employed to construct the initial molecular geometries and visualize the electron cloud distributions and spectral convolutions. The calculations were executed on high-performance computing resources provided by the National Center for High-Performance Computing (NCHC), Taiwan.

The simulation proceeded in three steps: (1) construction of molecular models, (2) optimization of the molecular structure, and (3) execution of the DFT simulation. The models of Alq3, Gaq3, and their respective molecular derivatives were first constructed. Their parameters were set by matching them with the required DFT simulation. Finally, the molecular models were optimized, and their properties were calculated.

### 2.2 Molecular Construction and Simulation Parameters

The initial structures of Alq3, Gaq3, and their respective derivatives were established using GaussView 4.1.2, where atomic coordinates were manually assigned to form chemically reasonable geometries. This involved adjusting bond lengths, bond angles, bond orders, and dihedral angles to reflect known coordination characteristics near the metal center and ligand framework. In particular, bond parameters around the C–H to N substitution sites were carefully tuned to ensure correct local geometry, and their values are summarized in Appendix Tables [Table tblA1] and [Table tblA2], which also compare the simulated values with experimental crystallographic data.^14,32^ These initial models served as the starting points for subsequent geometry optimization. During model construction, symmetrical alignment of the three ligand planes was applied to facilitate convergence. The structures were then optimized using energy minimization and atomic charge distribution criteria to obtain stable geometries. Figure 1 presents the optimized molecular structures of Alq3 and its seven derivatives along with a schematic of the ligand substitution strategy and corresponding abbreviations. In model construction, the symmetry of each ligand plane facilitated structural optimization. The optimization of the molecular structure was based on the atomic charge distribution. The molecular structure of Alq3 was constructed in GaussView 4.1.2; The names and abbreviations of the other derivatives are also listed in Figure 1. The molecular structures of Alq3 and its seven derivatives (Alq2p, Alc2p, Alz2p, Alx2p, Al-5n2p, Al-6n2p, and Al-7n2p) are shown with consistent abbreviations and are defined alongside their corresponding ligand names in the figure. Alq2p is Alq3 with one q ligand replaced by a p ligand, and the N atom is in position 1 (*n* = 1). The other six derivatives differ in the substitution position of the N atom (2 to 7), each corresponding to a unique ligand, as listed in the included table. The molecular structures of Gaq3 and its derivatives were constructed by replacing the central Al atom in the Alq3-based models with a Ga atom, followed by analogous structural adjustments.

**Figure 1:**
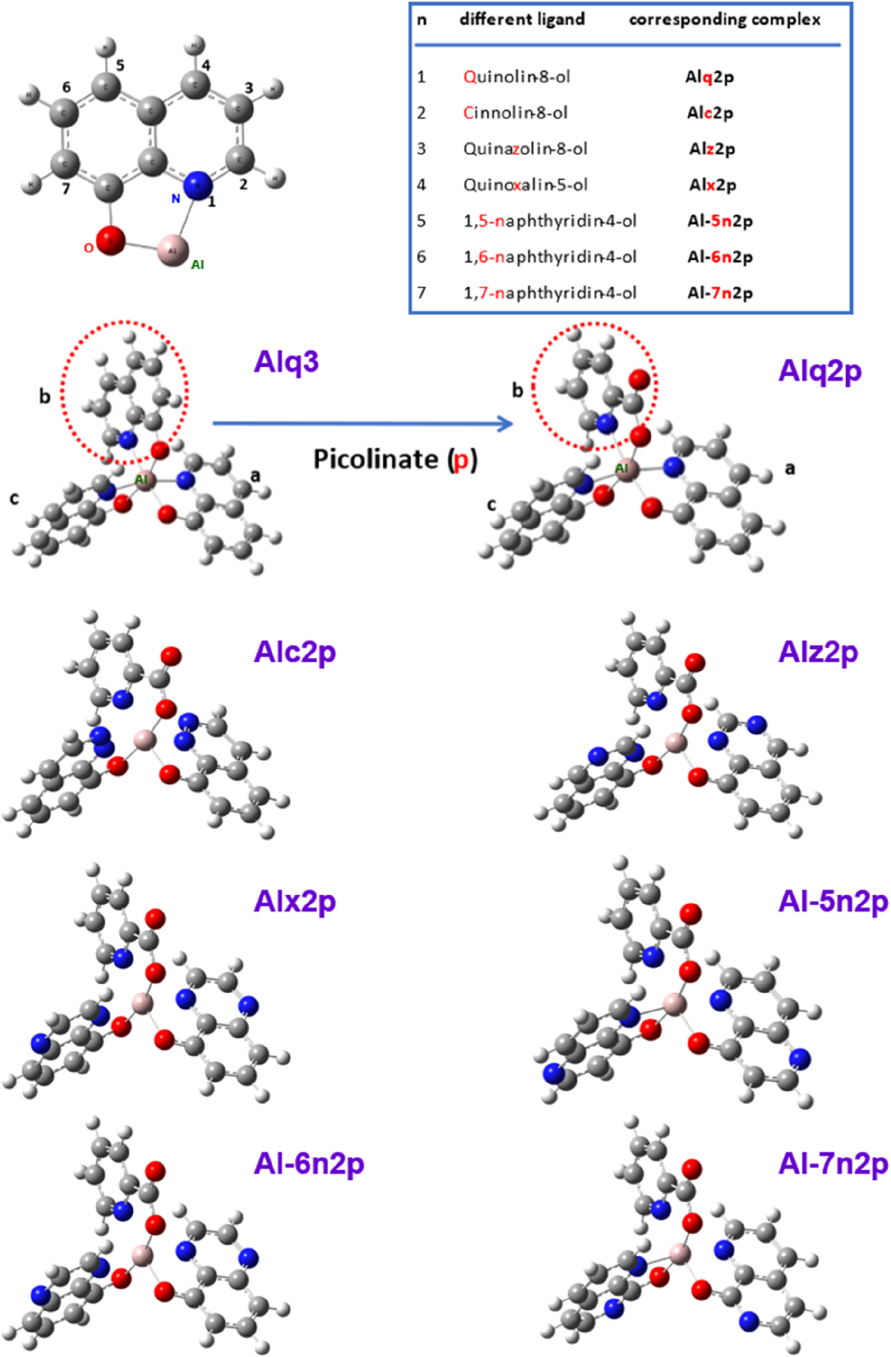
Molecular structure of Alq3 and its derivatives. The Al atom is pink. The O, N, C, and white atoms are red, blue, and white, respectively.

The DFT simulation proceeded in four steps: optimization, absorption energy calculation, excited-state optimization, and emission calculation. The job type, simulation methods, and basis set for each step are listed in Table 1. Step I, the geometric optimization of the molecular structure in the ground state, was performed using B3LYP with the 6–31G­(d) basis set. The vibrational frequencies were also calculated to obtain the optimized stable frequency of the structure. Step II was the calculation of the single-point absorption energy required for the transition from the ground state to the first excited state (S_1_). Step III was the calculation of the energy value that optimizes the structure in the first excited state. Step IV was the optimization of the structure of the excited state through a single-point energy calculation. For excited-state-related simulations (Steps II–IV), the 3–21G* basis set was employed. This choice is supported by prior literature,^18,32^ which demonstrated that TD-DFT calculations using the 3–21G* basis set yielded absorption spectra in close agreement with experimental observations for tris­(8-hydroxyquinolinato) metal complexes. Therefore, to balance structural accuracy and optical property prediction, the 6–31G­(d) and 3–21G* basis sets were respectively applied to ground- and excited-state calculations.

**Table 1:** Simulation Steps, Job Types, Simulation Methods, and Basis Functions

simulation step	job type	method	basis set
Step I. Ground-state optimization	opt + freq	DFT(B3LYP)	6–31G(d)
Step II. Absorption energy	energy	TD-DFT(B3LYP)	3–21G*
Step III. Excited-state structure optimization	opt	CIS	3–21G*
Step IV. Emission	energy	TD-DFT(B3LYP)	3–21G*

The parameter settings of the ground-state structure were used for each instance of optimization. In step I, DFT was used to optimize the ground-state structure; in step III, CIS computing was used. Because steps II and IV involved energy calculations, TD-DFT was used instead. The B3LYP exchange–correlation functions were all set to the 6–31G­(d) and 3–21G* basis sets. These functions were used in an approximate exchange method. In steps I and III, 6–31G­(d) and 3–21G* were used for structural optimization. All computational results for the electron cloud plots and spectral convolutions were obtained using the Gaussian 09 package and GaussView 4.1.2, respectively.

### 2.3 Validation against Experimental Data

To demonstrate the reliability of the present numerical framework, we compared the computed HOMO and LUMO energy levels of Alq_3_ and Gaq_3_ with experimental values,^33^ as illustrated in Figure 2. Although precise HOMO and LUMO values can vary with the chemical environment during measurement, the band gap is generally consistent and serves as a reliable reference point. Our Alq_3_ simulation predicted a 3.27 eV band gap (HOMO: −5.00 eV, LUMO: −1.73 eV), while experimental data show a 2.8 eV gap (HOMO: −5.8 eV, LUMO: −3.0 eV). Similarly, for Gaq_3_, our simulation yielded a 3.24 eV band gap (HOMO: −4.99 eV, LUMO: −1.75 eV), compared to the experimental 2.9 eV gap (HOMO: −6.3 eV, LUMO: −3.4 eV). While absolute energy values show slight variations, the predicted band gaps consistently follow the trend observed in the experimental data and remain within an acceptable range. This agreement supports the suitability of the chosen DFT method and reinforces the use of Alq_3_ and Gaq_3_ as reliable reference structures.

**Figure 2:**
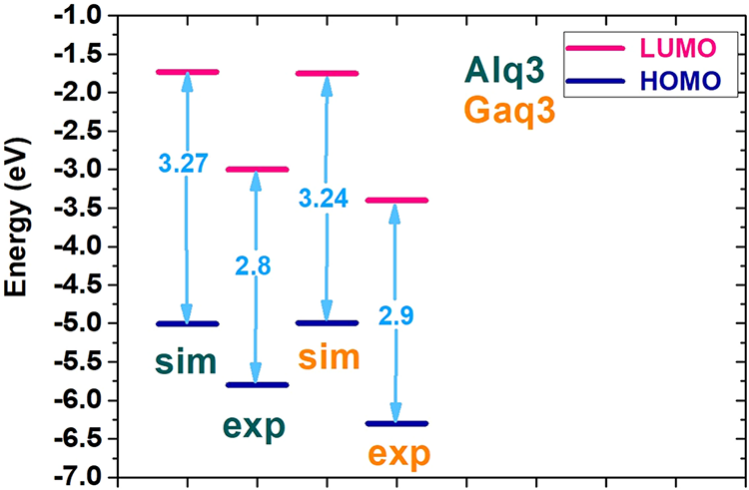
Comparison of simulated HOMO–LUMO levels and band gaps of Alq_3_ and Gaq_3_ with experimental data from Muhammad et al.^33^

## 3 Results and Discussion

### 3.1 Molecular Structure Optimization

Relative to Gaq3 and its derivatives, Alq3 and its derivatives had slight differences in molecular bonding but negligible differences in the relative positions of the atoms. Table 2 lists the total energy of each structure after optimization. The total energy of the ground state differed little among Alq3, Gaq3, and the derivatives. This indicated that the derivatives were stable in the simulation. For comparative clarity, the average ground-state energy of the Ga-centered molecules was −3341 ± 3.0 Ha, while that of the Al-centered molecules was −1661 ± 3.7 Ha, based on the values listed in Table 2. This trend suggests that Gaq3 derivatives possess relatively lower total energy, indicating a more stable molecular structure than the Al molecules.

**Table 2:** Total Energy after Optimization of Mlq3 (M = Al and Ga) and Derivatives (Denoted by the CH Group Substitution Position)

molecule	Alq3	Alq2p (1)	Alc2p (2)	Alz2p (3)	Alx2p (4)	Al-5n2p (5)	Al-6n2p (6)	Al-7n2p (7)
energy (Hartree)	–1671.9	–1631.7	–1663.7	–1663.8	–1663.7	–1663.8	–1663.7	–1663.8

molecule	Gaq3	Gaq2p (1)	Gac2p (2)	Gaz2p (3)	Gax2p (4)	Ga-5n2p (5)	Ga-6n2p (6)	Ga-7n2p (7)
energy (Hartree)	–3352.4	–3312.1	–3344.1	–3344.2	–3344.2	–3344.2	–3344.2	–3344.2

### 3.2 Dipole Moment

The dipole moment differed among the various CH/N substitution positions (Figure 3). In Figure 3, the horizontal axis presents the positions of the substituted CH groups, which, in turn, distinguish (and denote) the derivatives Alq2p, Alc2p, Alz2p, Alx2p, Al-5n2p, Al-6n2p, and Al-7n2p (positions 1 through 7, respectively). These derivatives were created by replacing the q_b_ ligand (quinoline) of Alq3 with a p ligand (pyridine). The positions of the CH group substitutions in the Alq3 and Gaq3 derivatives are given in Table 2. The dipole moment significantly increased from Alq3 to Alq2p (position 1) and then decreased from Alc2p (position 2) to Alz2p (position 3). When any of the q ligands of Alq3 was replaced with a p, the dipole moment significantly increased. Al-6n2p (position 6) had the highest dipole moment. The sequence of the Al molecules ordered by dipole moment was Al-6n2p (6) > Al-7n2p (7) > Al-5n2p (5) > Alc2p (2) > Alx2p (4) > Alz2p (3) > Alq2p (1) > Alq3. The trend in the dipole moments of Gaq3 and its derivatives was similar to that of Alq3 and its derivatives. Although the dipole moments of Ga-6n2p and Ga-7n2p were slightly smaller than those of Al-6n2p and Al-7n2p, the dipole moments of the other Ga molecular structures were slightly larger than those of the Al molecular structures because, relative to Al, Ga has a larger atomic weight, and its nucleus thus attracts electrons more strongly. Therefore, the valence electrons were closer to the Ga atom than to the Al atom, leading to greater polarization.

**Figure 3:**
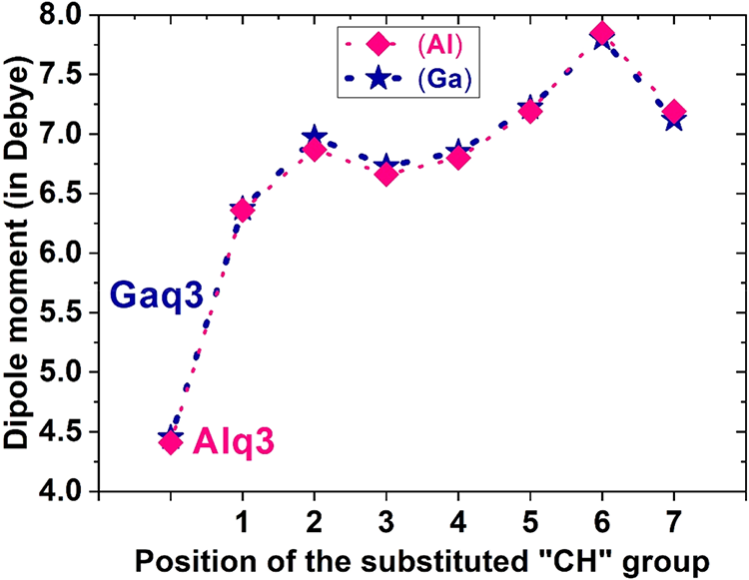
Plot of the dipole moment at different CH/N-substituted positions. Positions 1 to 7 represent 1:Alq2p, 2:Alc2p, 3:Alz2p, 4:Alx2p, 5:Al-5n2p, 6:Al-6n2p, and 7:Al-7n2p, respectively.

### 3.3 Spectra

The electronic excitation energy can be obtained by using TD-DFT and electron cloud calculations. The absorption and emission spectra were also calculated. The peak absorption and emission wavelengths are shown in Figure 4(a,b), respectively, for the Mq3 (M = Al and Ga) molecules and their derivatives; Tables 3 and 4 list the values. To provide a more intuitive understanding of their photophysical behavior, the full simulated absorption spectra of Alq3, Alq2p, and their derivatives, as well as Gaq3, Gaq2p, and their derivatives, are provided in Figure [Fig figA1](a,b), showing their wavelength-dependent absorption intensities. Both the absorption and emission spectra of Alq3 and its derivatives were similar to those of Gaq3 and its derivatives. As presented in Figure 4(a,b), the position of the CH/N substitution affected both the peak absorption and emission wavelengths and thus can be used to tune the color of the OLED materials. The peak absorption wavelength can be tuned between 370.02 and 478.46 nm for the Al molecules and between 370.73 and 478.64 nm for the Ga molecules. The peak emission wavelength can be changed between 427.59 and 646.77 nm for the Al molecules and between 429.15 and 653.65 nm for the Ga molecules.Among the Al derivatives, Alq2p (*n* = 1) was red-shifted from Alq3 by approximately 9 nm. The largest red shift was for Alx2p (*n* = 4) at 114 nm, followed by Alc2p (*n* = 2) at 68 nm, and the largest blue shift was for Al-5n2p (*n* = 5) at 105 nm, followed by Al-7n2p (*n* = 7) at 48 nm. Among the Ga derivatives, Gaq2p (*n* = 1) and Gaq3 had radiation red shifts of approximately 7 nm. The largest red shift was for Gax2p (*n* = 4) at 117 nm, followed by Gac2p (*n* = 2) at 74 nm, and the largest blue shift was for Ga-5n2p (*n* = 5) at 107 nm, followed by Ga-7n2p (*n* = 7) at 46 nm. The Al and Ga derivatives had similar relationships between their peak absorption and emission wavelengths at the CH/N substitution position. In addition, the Ga derivatives had red shifts that were 2–9 nm higher than those of Al derivatives. This observation accords with those in the literature.^11^ The values of the Stokes shift were calculated (Tables 3 and 4) from the gap between the absorption and emission peaks in the spectra. For example, the energy of the absorption peak of Al-5n2p was higher than that of the Al-5n2p emission peak. Thus, the energy of the emission photon is lower than that of the absorption photon. The Stokes shift value is the difference between the emission and absorption energy. Our optimized organic molecular structures were such that the ground state and excited state differed little except for some changes in the atomic bonds. Comparing the results in Figure 4(a,b) or those in Tables 3 and 4, we found that the dependence of the absorption wavelength on the substitution position was similar to that of the emission wavelength. This phenomenon is known as mirror symmetry in organic materials.

**Table 3:** Wavelengths of Absorption and Emission Peaks and Stokes Shift Values of Alq3 and Its Derivatives

	absorption wavelength (nm)	emission wavelength (nm)	stokes shift (nm)
Alq3	423.08	524.13	101.05
Alq2p	426.96	532.73	105.77
Alc2p	454.41	600.99	146.58
Alz2p	428.15	554.81	126.66
Alx2p	478.46	646.77	168.31
Al-5n2p	370.02	427.59	57.57
Al-6n2p	432.89	535.62	102.73
Al-7n2p	398.02	485.06	87.04

**Table 4:** Wavelengths of Absorption and Emission Peaks and Stokes Shift Values of Gaq3 and Its Derivatives

	absorption wavelength (nm)	emission wavelength (nm)	stokes shift (nm)
Gaq3	425.25	529.23	103.98
Gaq2p	428.44	536.16	107.72
Gac2p	457.91	609.85	151.94
Gaz2p	431.91	561.42	129.51
Gax2p	478.64	653.65	175.01
Ga-5n2p	370.73	429.15	58.42
Ga-6n2p	436.76	540.79	104.03
Ga-7n2p	399.91	489.70	89.79

**Figure 4:**
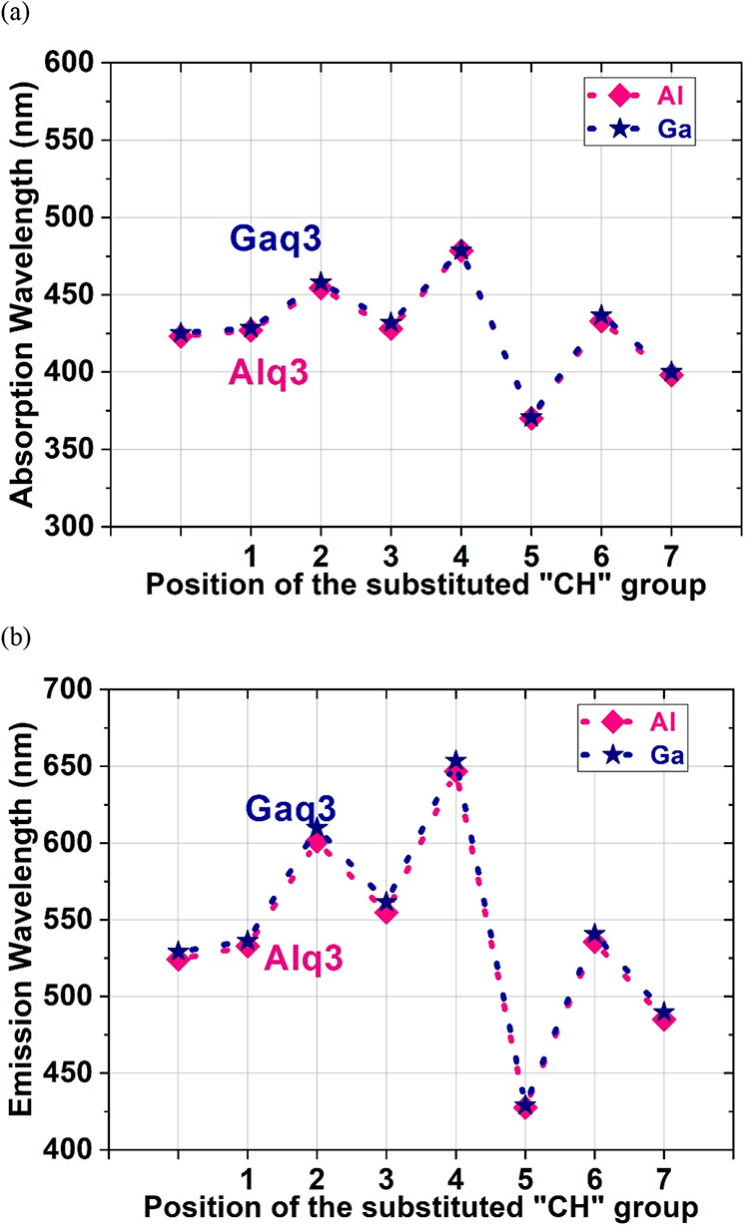
Peak (a) absorption and (b) emission wavelengths for Mq3 (M = Al, Ga) and their derivatives.

### 3.4 Band Gap

To better understand the electronic structure variations induced by ligand modifications and CH/N substitution, the HOMO and LUMO energy levels and the corresponding band gaps are summarized in Table 5. These numerical results provide a foundation for analyzing the trends in energy level shifts and band gap modulation. In the following discussion, these data are visualized and further interpreted through the energy level diagrams shown in Figure 5, which illustrate the energy level and band gap of Alq3 and its derivatives created by replacing the q_b_ ligand (quinoline) with a p ligand (picolinate). Compared to Alq2p, each of the six CH/N-substituted derivatives contains two additional nitrogen atoms, introduced through substitution of CH groups. These substitutions lead to a simultaneous reduction in both HOMO and LUMO energy levels. The energy levels of these derivatives were position-dependent. The HOMO and LUMO energies of the derivatives were significantly lower than those of Alq3. In particular, Alx2p and Al-5n2p had the lowest LUMO and HOMO energies, respectively. Compared with Alq_2_p, all six derivatives exhibit a simultaneous decrease in HOMO and LUMO energy levels due to the addition of two nitrogen atoms. CH substitution significantly impacts electronic properties, resulting in a greater energy level reduction in these derivatives compared with Alq_3_ and Alq_2_p. Specifically, Al-5n2p exhibits the largest HOMO energy decrease (0.783 eV relative to Alq_2_p), while Alc2p shows the smallest reduction (0.313 eV relative to Alq_2_p). Similarly, in terms of the LUMO energy level, the most significant reduction occurs in Alx2p, showing a decrease of 0.462 eV compared to the LUMO of Alq_2_p. In contrast, the smallest reduction is observed in Al-7n2p, with a decrease of 0.207 eV relative to that in Alq_2_p. Thus, the molecular structures of these derivatives were more stable than that of Alq3. Specifically, external electrons could be easily injected through the conduction band, resulting in high electron injection efficiency. The energy level stability and electron injection efficiency of all Alq3 derivatives were higher than those of Alq3.

**Figure 5:**
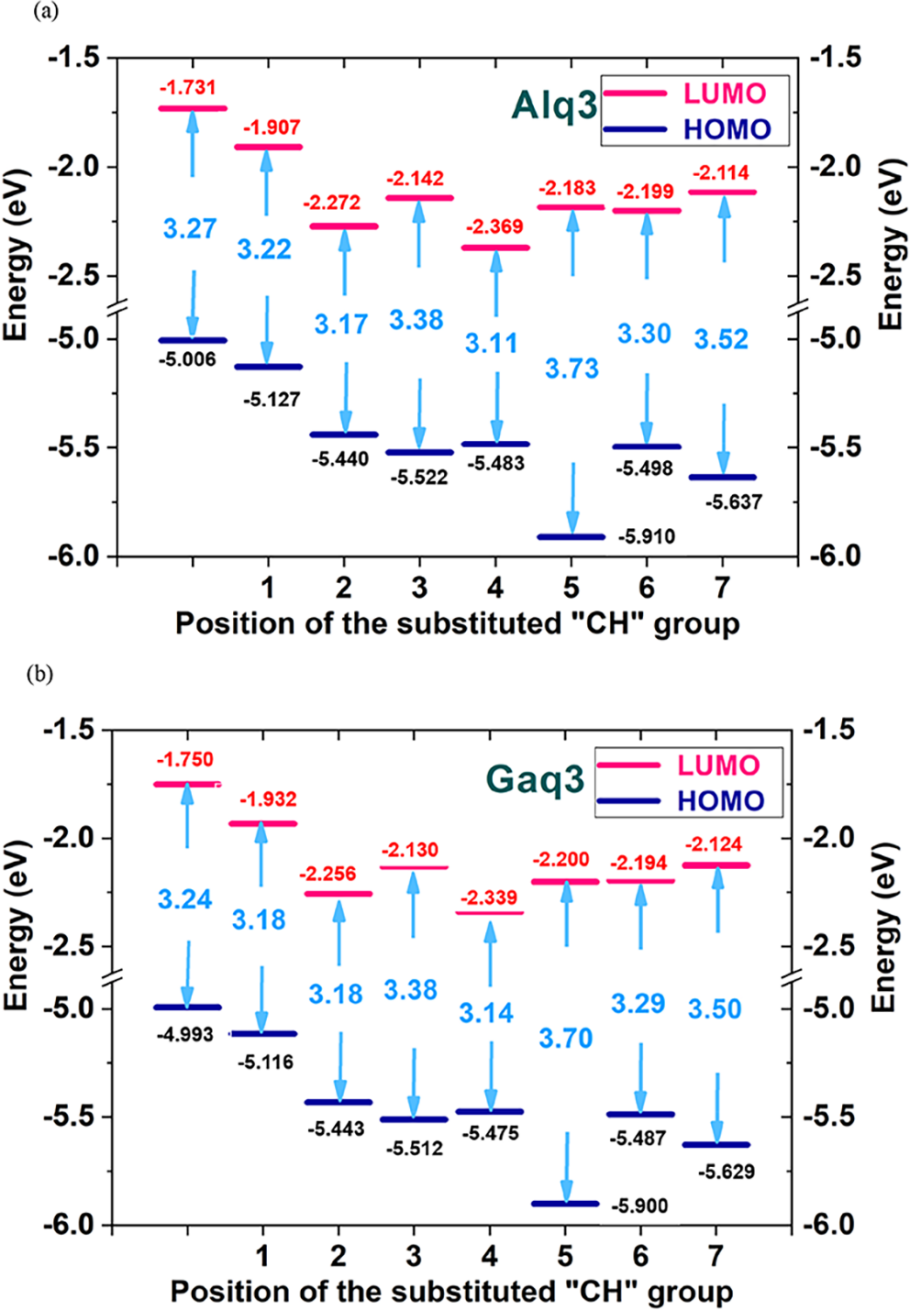
Energy level and band gap of (a) Alq3 and derivatives and (b) Gaq3 and derivatives.

Figure 5(b) illustrates the energy level and band gap of Gaq3 and its derivatives; the results are similar to those of Alq3 and its derivatives. As shown in Figure 5(b), from Gaq3 to Gaq2p, the HOMO and LUMO energies decreased by 0.123 and 0.182 eV, respectively. When comparing Gaq_2_p and its derivatives, variations in energy levels are observed due to CH substitutions. Ga-5n2p exhibited the largest HOMO energy reduction (0.784 eV), while Gac2p had the smallest (0.317 eV). For LUMO energy, Gax2p showed the greatest reduction (0.407 eV), and Ga-7n2p showed the least reduction (0.192 eV). These results suggest that the electronic properties of Gaq_2_p derivatives can be fine-tuned through CH substitution, influencing both molecular stability and electron transport efficiency.

**Table 5:** HOMO and LUMO Energy Levels and Energy Gaps of Mq3 and Mq2p (M = Al, Ga), and Their Derivatives

complex	HOMO (eV)	LUMO (eV)	*E*_g_ (eV)	complex	HOMO (eV)	LUMO (eV)	*E*_g_ (eV)
Alq3	–5.006	–1.731	3.274	Gaq3	–4.993	–1.750	3.243
Alq2p	–5.127	–1.907	3.219	Gaq2p	–5.116	–1.932	3.184
Alc2p	–5.440	–2.272	3.168	Gac2p	–5.433	–2.256	3.177
Alz2p	–5.522	–2.142	3.379	Gaz2p	–5.512	–2.130	3.382
Alx2p	–5.483	–2.369	3.114	Gax2p	–5.475	–2.339	3.136
Al-5n2p	–5.910	–2.183	3.727	Ga-5n2p	–5.900	–2.200	3.699
Al-6n2p	–5.498	–2.199	3.298	Ga-6n2p	–5.487	–2.194	3.293
Al-7n2p	–5.637	–2.114	3.522	Ga-7n2p	–5.629	–2.124	3.504

Figure 6(a) illustrates the electron clouds of Alq3 and its derivatives. In the HOMO diagrams, the electron clouds for the eight derivatives are the same on the qa ligand. Because the phenoxide ring had a CH/N substitution, the electron clouds were more concentrated around the phenoxide ring (O11, C9, C8, C7, and C5). The sequence of Al molecules in descending order of HOMO stability was Al-5n2p > Al-7n2p > Alz2p > Al-6n2p > Alx2p > Alc2p > Alq2p > Alq3. With respect to the LUMO, the electron cloud distributions differed between the molecular structures as follows. When the CH/N substitution was in the pyridyl ring (Alc2p, Alz2p, and Alx2p), the electron cloud was primarily concentrated on the pyridyl ring of the qc ligand. Furthermore, when the CH/N substitution was in the phenoxide ring (Al-5n2p, Al-6n2p, and Al-7n2p), the electron cloud was primarily concentrated on the p ligand. The sequence of Al molecules ordered by overall LUMO stability was Alx2p > Alc2p > Al-6n2p > Al-5n2p > Alz2p > Al-7n2p > Alq2p > Alq3.

**Figure 6:**
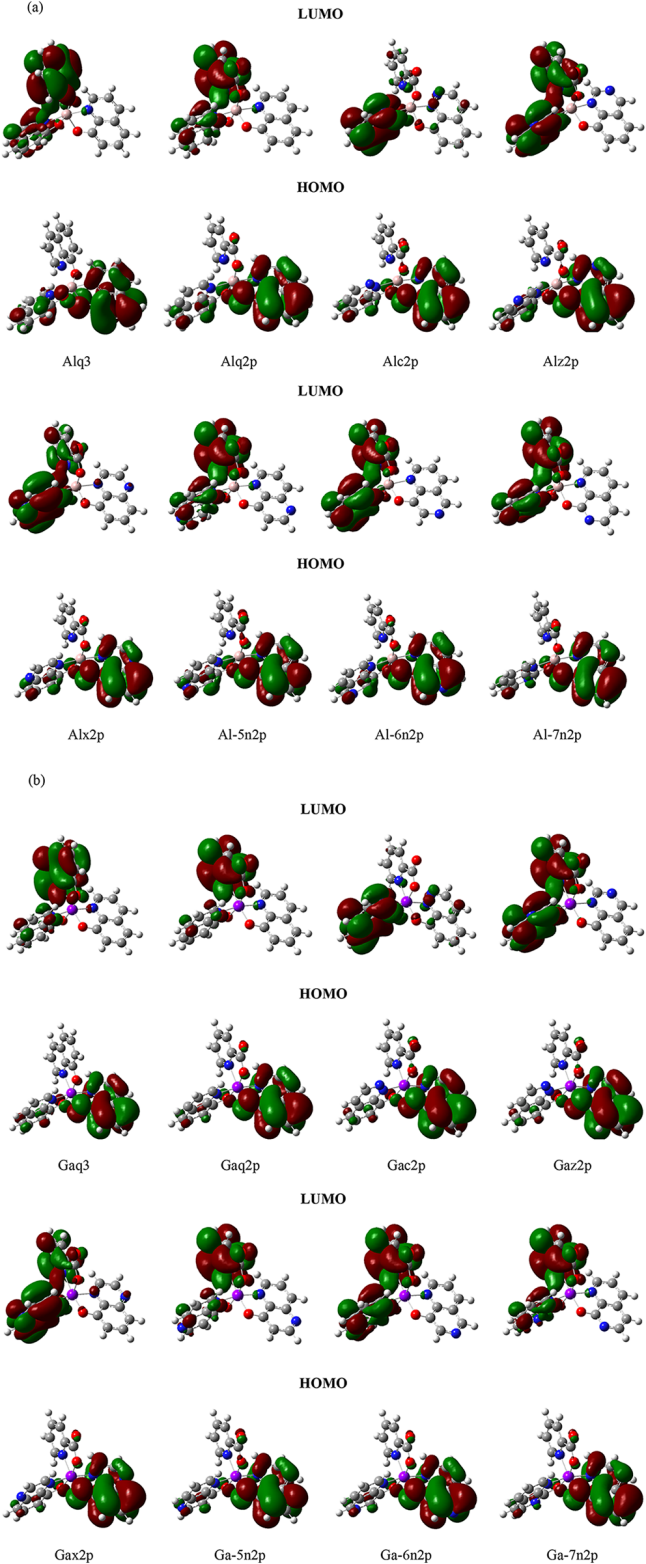
Electron clouds of (a) Alq3 and Alq2p and their derivatives and (b) Gaq3 and Gaq2p and their derivatives.

Figure 6(b) illustrates the electron clouds of Gaq3 and its derivatives. The HOMO findings were similar to those of Alq3 and its derivatives. When the CH/N substitution was in the phenoxide ring, the electron cloud was concentrated on the phenoxide ring. The sequence of derivatives ordered by HOMO stability was Ga-5n2p > Ga-7n2p > Gaz2p > Ga-6n2p > Gax2p > Gac2p > Gaq2p > Gaq3. The difference between the Ga and Al electron cloud trends was that Gaz2p was more stable than Ga-6n2p. The electron cloud and LUMO findings were also similar to those of Al. The trend of the Gaq3 derivatives with respect to LUMO stability was the same as that for the Al molecules, save for Ga-5n2p being more stable than Ga-6n2p.

Although the electron cloud findings were similar between the Alq3 and Gaq3 derivatives, the HOMOs of the Gaq3 derivatives were more concentrated on the qa ligand. This redistribution resulted in a slight increase in HOMO energy, which in turn affected the work function alignment with the anode.^34^ The enhanced orbital overlap led to better electron cloud matching, which in turn facilitated hole regeneration, as the HOMO level governs the hole transport properties in organic semiconductors.^35,36^

### 3.5 Ionization Potential and Electron Affinity

A conceptual explanation of the ionization potential (IP) and electron affinity (EA) is provided here to clarify their physical meaning and computational relevance. The IP is defined as the energy required to remove an electron from an atom, ion, or molecule to an infinite distance, reflecting the material’s ability to block holes. Conversely, electron affinity represents the energy change when an electron is added to a neutral atom or molecule, forming a monovalent negative ion, and is related to the electron injection capability. The ionization potential and electron affinity of the Al and Ga molecules are shown in Figure 7(a,b), respectively. The ionization potential and electron affinity of Alq3 and Ga derivatives differ notably from those of the original Alq3 and Gaq3 molecules. The higher ionization energies of the derivatives indicate stronger hole-blocking ability, as materials with deeper HOMO levels can better prevent hole leakage. Meanwhile, the higher electron affinities observed in Alq2p, Gaq2p, and their respective derivatives reflect an improved electron injection efficiency. These trends reflect favorable shifts in HOMO and LUMO energy level shifts induced by ligand modification, which enhance alignment with adjacent layers and thereby facilitate more efficient charge transfer.

**Figure 7:**
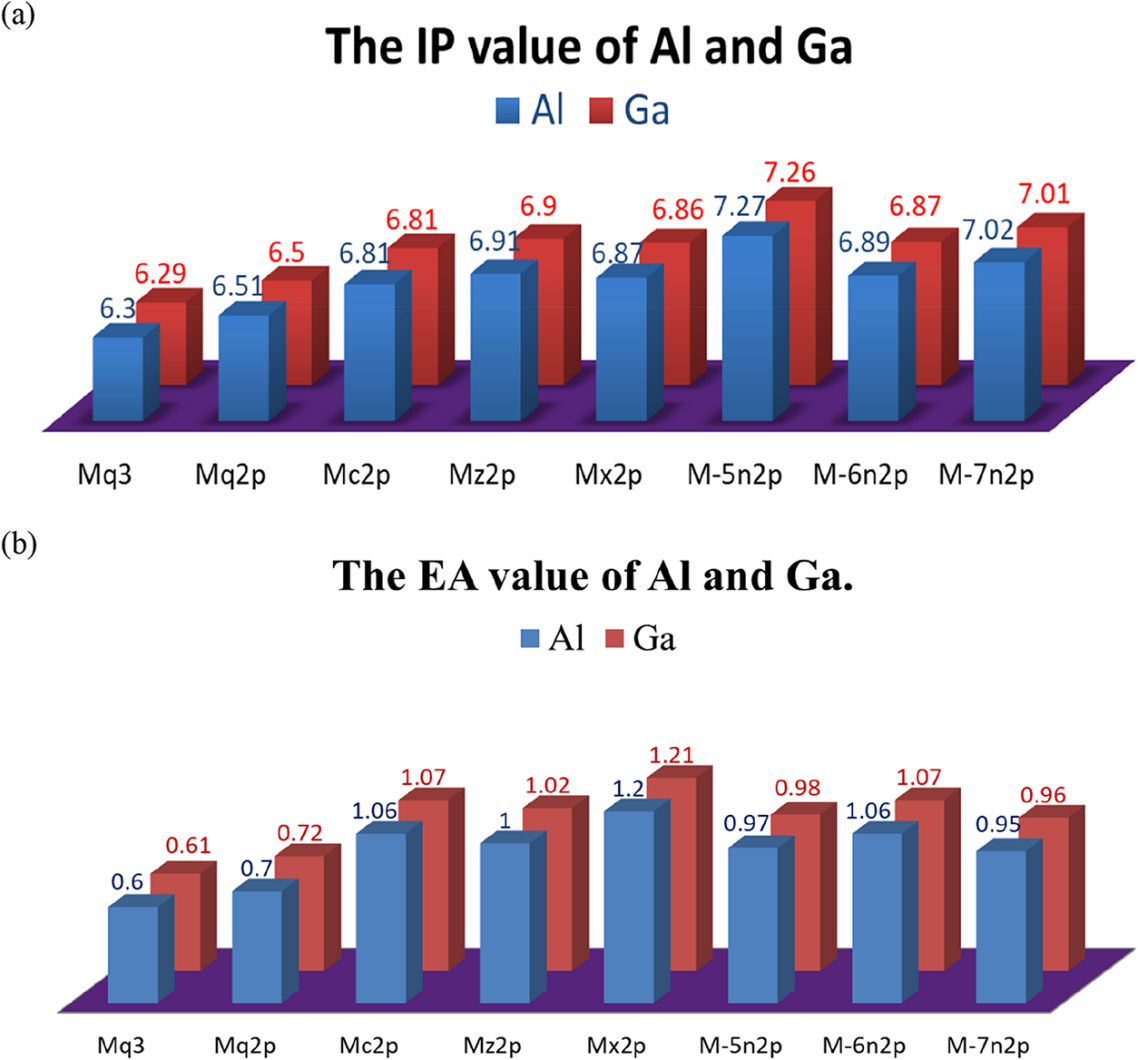
(a) Ionization energy and (b) electron affinity of Al and Ga molecules and their derivatives.

Therefore, the hole-blocking ability of all derivatives was superior to that of Alq3. The electron affinity of Alq2p and its derivatives was greater than that of Alq3. Consequently, the electron injection efficiency of Alq2p can be improved beyond that of Alq3 through electron injection from the electrodes into the material. The trends for the ionization energy and electron affinity of the Ga molecules were the same as those for the Al molecules. Therefore, the hole-blocking ability and electron injection efficiency of Gaq2p and its derivatives were superior to those of the original Gaq3.

In addition to frontier orbital energy levels, charge transport properties play a crucial role in determining the efficiency of the OLED. To further assess the charge carrier mobility, reorganization energies (λ_h_ for holes and λ_e_ for electrons) were calculated based on the Marcus electron transfer theory,^37^ as they are key parameters governing charge transfer rates. According to this theory, the charge transfer rate constant *K*_h/e_ for holes (h) or electrons (e) is given by

1 $$K_{h/e}={\left(\frac{\pi }{\lambda_{h/e}\mathrm{kT}}\right)}^{1/2}\times \frac{V_{h/e}^{2}}{\hbar }\times \exp\left(-\frac{\lambda_{h/e}}{4\mathrm{kT}}\right)$$

where *k* is the Boltzmann constant, ℏ is the reduced Planck constant, and *T* is the temperature.

The rate is primarily influenced by the electronic coupling term *V*_h/e_ and the reorganization energy λ_h/e_. Since *V*_h/e_ values across the derivatives vary minimally, the reorganization energy becomes the dominant factor affecting charge mobility. The use of reorganization energy as a reliable descriptor for evaluating charge transport in OLED materials has also been emphasized in previous studies.^38,39^ Table 6 presents the calculated reorganization energies for both hole (λ_h_) and electron (λ_e_) transport in Alq3, Gaq3, and their respective derivatives. For Al-based molecules, Alq2p shows a higher λ_e_ than Alq3, suggesting slower electron transport. In contrast, all Alq2p derivatives exhibit reduced λ_e_ values compared to those of Alq2p, indicating enhanced electron mobility. A similar trend is found in the Ga-based series, where Gaq2p has a larger λ_e_ than Gaq3, but its derivatives (e.g., Gaz2p, Gax2p, and Ga-6n2p) have lower λ_e_, some even surpassing the parent Gaq3 in charge transport potential. Moreover, most Ga derivatives have smaller λ_e_ values than their Al counterparts, reinforcing Gaq3′s superiority as a charge-transporting material, which partly explains its popularity in advanced OLED applications.

**Table 6:** Calculated Hole (λ_h_) and Electron (λ_e_) Reorganization Energies for Alq3, Gaq3, and Their Derivatives

	Alq3	Alq2p	Alc2p	Alz2p	Alx2p	Al-5n2p	Al-6n2p	Al-7n2p
λ_h_(eV)	0.21	0.27	0.26	0.28	0.29	0.26	0.29	0.26
λ_e_(eV)	0.25	0.33	0.25	0.24	0.25	0.32	0.26	0.31

	Gaq3	Gaq2p	Gac2p	Gaz2p	Gax2p	Ga-5n2p	Ga-6n2p	Ga-7n2p
λ_h_(eV)	0.19	0.25	0.26	0.27	0.27	0.25	0.27	0.25
λ_e_(eV)	0.24	0.32	0.24	0.19	0.20	0.30	0.19	0.27

## 4 Conclusions

This study optimized the structure of Mq3 and Mq2p (M = Al, Ga) and their derivatives through density functional theory (DFT) calculations. Key properties, such as total energy of the ground state, dipole moment, absorption and emission wavelengths, HOMO and LUMO levels, ionization potential, and electron affinity, were systematically evaluated. The main findings are summarized as follows:

- Ground-state total energy: Gaq_3_ and its derivatives exhibit lower total energies compared to Alq_3_ and its derivatives, suggesting higher thermodynamic stability for Ga-based complexes.
- Dipole moment: Gaq3 and its derivatives exhibit higher dipole moments than Alq3 analogues, primarily due to increased molecular asymmetry from q_b_-to-p ligand substitution.
- Absorption and emission wavelengths: Alq_3_ and Gaq_3_ derivatives exhibited tunable absorption and emission spectra. CH/N substitution induced position-dependent spectral shifts, with Ga derivatives showing slightly greater red shifts, enabling emission color tuning via ligand modification.
- HOMO and LUMO energies: CH/N substitution led to significant reductions in HOMO and LUMO energy levels for both Alq_3_ and Gaq_3_ derivatives. Alx_2_p and Gax_2_p showed the lowest LUMO levels, while Al-5n_2_p and Ga-5n_2_p exhibited the deepest HOMO levels, enhancing molecular stability and charge transfer potential.
- Ionization potential and electron affinity: Alq2p and Gaq2p derivatives exhibit higher ionization potentials and electron affinities than their parent compounds (Alq3 and Gaq3), indicating enhanced hole-blocking ability and improved electron injection efficiency due to better frontier orbital alignment.

Overall, this study provides useful insights into the electronic and optical properties of Alq3 and Gaq3 derivatives, making them promising for both emissive and electron transport layers in the form of OLEDs. Gaq3 derivatives also offer greater stability and performance potential.

## Figures and Tables

**A1 figA1:**
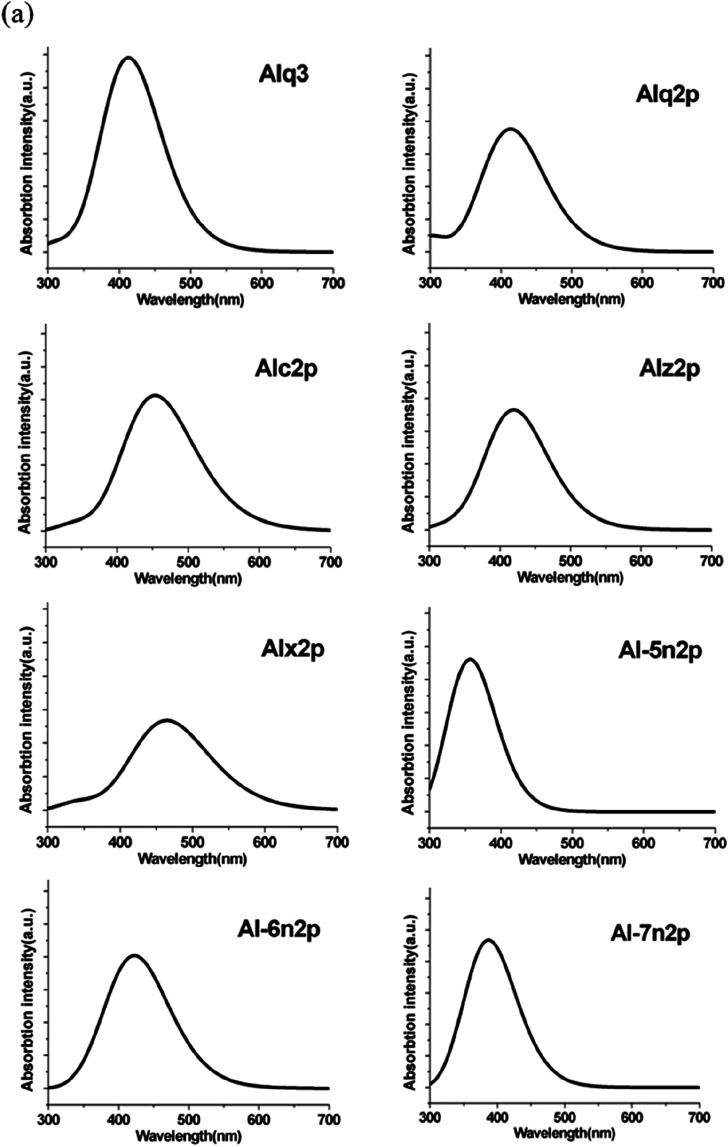

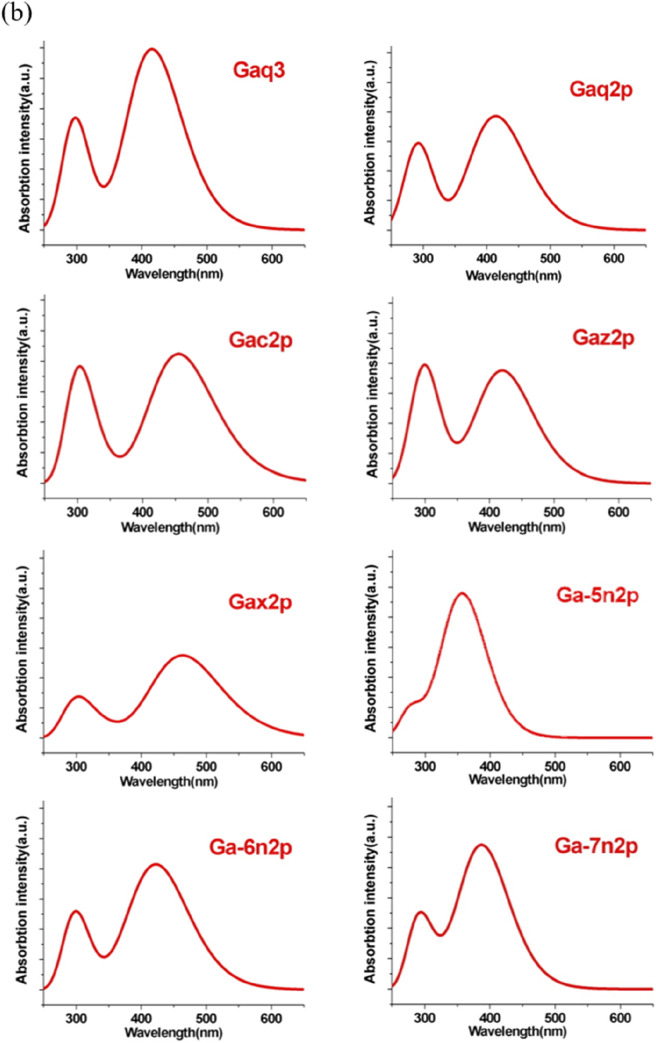
Simulated absorption spectra of (a) Alq3, Alq2p, and their derivatives; (b) Gaq3, Gaq2p, and their derivatives, obtained from TD-DFT calculations.

## Appendix

See Figure [Fig figA1], Tables [Table tblA1] and [Table tblA2].

**A1 tblA1:** Calculated Bond Lengths and Bond Angles of Alq3, Alq2p, and their Derivatives Compared with Experimental Data^1^

R(Ǻ) & φ(°)	Alq3	Alq2p	Alc2p	Alz2p	Alx2p	Al-5n2p	Al-6n2p	Al-7n2p	exp.^1^
Al–N (Ǻ)	2.083	2.083	2.073	2.090	2.093	2.082	2.091	2.086	2.050
Al–O (Ǻ)	1.855	1.848	1.869	1.855	1.849	1.862	1.853	1.852	1.850
Na–Al–O (°)	83.36	83.54	82.87	83.48	83.49	83.63	83.80	83.35	83.63

**A2 tblA2:** Calculated Bond Lengths and Bond Angles of Gaq3, Gaq2p, and their Derivatives Compared with Experimental Data^2^

R(Ǻ) & φ(°)	Gaq3	Gaq2p	Gac2p	Gaz2p	Gax2p	Ga-5n2p	Ga-6n2p	Ga-7n2p	exp.^2^
Ga–N (Ǻ)	2.096	2.095	2.084	2.100	2.108	2.094	2.105	2.097	2.093
Ga–O (Ǻ)	1.934	1.928	1.946	1.934	1.927	1.941	1.932	1.932	1.937
Na–Ga–O (°)	81.92	82.11	81.52	82.14	82.10	82.18	82.33	81.85	82.0
